# SFA-Net: Scale and Feature Aggregate Network for Retinal Vessel Segmentation

**DOI:** 10.1155/2022/4695136

**Published:** 2022-10-21

**Authors:** Jiajia Ni, Jinhui Liu, Xuefei Li, Zhengming Chen

**Affiliations:** College of Internet of Things Engineering, Hohai University, Changzhou, China

## Abstract

A U-Net-based network has achieved competitive performance in retinal vessel segmentation. Previous work has focused on using multilevel high-level features to improve segmentation accuracy but has ignored the importance of shallow-level features. In addition, multiple upsampling and convolution operations may destroy the semantic feature information contained in the decoder layer. To address these problems, we propose a scale and feature aggregate network (SFA-Net), which can make full use of multiscale high-level feature information and shallow features. In this paper, a residual atrous spatial feature aggregate block (RASF) is embedded at the end of the encoder to learn multiscale information. Furthermore, an attentional feature module (AFF) is proposed to enhance the effective fusion between shallow and high-level features. In addition, we designed the multi-path feature fusion (MPF) block to fuse high-level features of different decoder layers, which aims to learn the relationship between the high-level features of different paths and alleviate the information loss. We apply the network to the three benchmark datasets (DRIVE, STARE, and CHASE_DB1) and compare them with the other current state-of-the-art methods. The experimental results demonstrated that the proposed SFA-Net performs effectively, indicating that the network is suitable for processing some complex medical images.

## 1. Introduction

**Figure 1:**
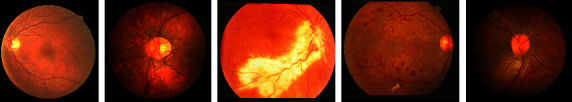
.Various fundus retinal data images containing the disease.

Retinal vessel image segmentation is an important medical image processing method for diagnosing and treating common eye diseases such as high blood pressure [1], as shown in Figure 1. However, the retinal vessels have a complex structure and a small area of interest compared to other medical images, making manual segmentation time-consuming and limited by expert experience [2]. Therefore, in order to tackle the problem, it is necessary to propose an automatic segmentation algorithm for retinal vessel segmentation.

With the development of artificial intelligence, many different efforts have been dedicated to segmenting retinal vessel images [1, 3–7]. It can be broadly divided into traditional machine learning segmentation methods and deep learning methods. Traditional methods focused on adopting hand-crafted features to segment retinal blood vessels. For instance, fully-connected conditional random field (FCCRF) is used to segment retinal vessel images [8]. Similarly, Orlando et al. [9] also used FCCRF to segment the retinal vessels. Sreejini and Govindan [10] employ particle swarm optimization (PSO) to optimally filter parameters of the multiscale Gaussian matched filter (MF) for improving the accuracy of retina vessel segmentation. After that, fuzzy C-means are employed to segment retinal blood vessels [11]. Although these traditional machine learning approaches show good performance in some cases, hand-crafted features [12, 13] depend much too heavily on prior knowledge and, hence, fail in datasets with many complex cases. Due to the powerful feature extraction capability of deep learning, semantic segmentation tasks using deep learning are gradually becoming mainstream. Therefore, we used deep learning for the retinal vessel segmentation.

In recent years, people have begun to use convolutional neural networks for semantic segmentation. For example, Dasgupta and Singh [14] and Mo and Zhang [15] employ fully convolutional networks (FCN) to segment retinal blood vessels and achieve good performance. Later, U-Net [16] and many U-shaped structure-based networks [6, 17–19] have been proposed, which significantly improve the semantic segmentation accuracy. For instance, Khan et al. [20, 21] developed a hybrid deep learning model that combines the DenseNet and the U-Net for semantic segmentation. Although the algorithms perform well in semantic segmentation, they do not consider the problem of feature information loss in the encoding and decoding stages. U-Net++ [17] designed a densely connected coding-decoding structure in order to improve semantic segmentation accuracy and reduce the problem of feature information loss. However, the U-Net++ has a complex structure and too many training parameters, which are prone to overfitting. SCS-Net [22] also used an encoder-decoder structure to fundus vascular segmentation, considering the problem of information loss in the decoding phase but ignoring the same problem in the encoding phase. To a certain extent, these methods are dependent on the encoder-decoder structure, which focuses on processing the feature maps obtained from the feature samples of the decoder and ignores shallow features. The shortcomings of those methods are that they have not been good enough for feature extraction from the encoding and decoding layers.

To address the aforementioned problems, we propose a new framework that follows the pipeline of encoder-decoder. In the decoding layer, a new module is designed to make better use of the output features of the different layers in the decoding stage. It solves the phenomenon of feature loss in the feature decoding stage. In addition, we also redesigned two feature extraction modules to solve the problem of feature loss in the encoding stage. These two modules make full use of the features at different scales and different layers to supplement the feature information lost in the coding stage. Herein, we propose a scale and feature aggregate network for retinal vessel segmentation (SFA-Net), which is an encoder-decoder structure with three basic components. First, the residual atrous spatial feature aggregate (RASF) module is set at the end of the encoder layer and is used to effectively learn multiscale feature information. Then, in the decoder layer, the vanilla skip connection is replaced by an attention feature fusion (AFF) module. Finally, a multi-path feature fusion (MPF) module is designed to learn the high-low features of different stages. In addition, in the MPF module, we introduce an attention mechanism that can effectively suppress noise and redundant information. In summary, we make the following key contributions in this paper:

1. We propose to use the RASF block to learn multiscale feature information and increase the receptive field of the network.
2. To adaptively combine high-level features information with shallow features information, the attention feature fusion (AFF) module is introduced. Then, we apply an MPF module to extract more global semantic information from high-level features.
3. Based on the RASF, AFF, and MPF blocks. We propose an efficient retinal vessel segmentation model (SFA-Net) and teste it in different fundus datasets. The experimental results demonstrate that our model can effectively improve the vessel segmentation results

## 2. Related Work

### 2.1. Image Semantic Segmentation

The main current semantic segmentation methods are usually based on deep learning [16, 19, 23–26]. A fully convolutional network (FCN) [26] is the first end-to-end neural network to be applied to semantic segmentation. Later, many FCN-based semantic segmentation networks have been proposed. For example, UI-Net [27] runs the FCN model several times and builds upon a state-of-the-art FCN as an active user model for training. Drozdzal et al. [28] combined FCN with fully convolutional residual networks (FC-ResNets) for medical image segmentation. Another famous semantic segmentation network is U-Net [16], which is a classical encoder-decoder structure. U-Net introduced vanilla skip connections to connect the high-level features and shadow features in the encoding and decoding layers. Inspired by U-Net, many U-shaped-based structure networks have been proposed for semantic segmentation [29–33]. Later, with rapid advancements in convolutional neural network technology, such as multiscale context extraction and attention mechanisms, were introduced to the semantic segmentation task. For instance, the DeepLab family [29, 30] proposed the atrous spatial pyramid pooling (ASPP) block to capture multiscale features by increasing the network receptive field. The pyramid pooling module was used by PSPNet [34] to improve the network's capacity to utilize global context information. Woo et al. [35] introduced channel attention and spatial attention for adaptive feature refinement. ATTU-Net [23] proposed an attention gate (AG) model to learn target structures of varying shapes and sizes. Dual attention [36] uses spatial and channel attention to capture abundant contextual features to better improve the network's ability to segment the blood vessels in the fundus. We also use multiscale feature extraction and attention mechanisms in our network.

### 2.2. Retinal Vessel Segmentation

There are many different algorithms for retinal vessel segmentation, and these can be divided into two main categories: traditional machine learning methods and deep learning methods. Traditional machine learning segmentation methods utilize hand-crafted features to conduct retinal vessel segmentation. Such as fuzzy C-means [11] is employed to segment retinal blood vessels. Mahapatra et al. [37] proposed a novel framework for retinal vessel segmentation using an optimal improved frangi filter and adaptive weighted spatial fuzzy C-means. Zhang et al. [38] used an unsupervised texton dictionary, where vessel textons were derived from the responses of a multiscale Gabor filter bank. Lupascu et al. [39] adopted a feature-based AdaBoost classifier for segmenting retinal vessels. Javidi et al. [40] and Zhang et al. [41] used sparse coding for retinal vessel enhancement and segmentation. However, traditional methods rely on manually extracted features, and their accuracy is difficult to guarantee.

Following the successful application of convolutional neural networks (CNNs) in the medical image field, many CNN-based segmentation networks have been applied to retinal vessel segmentation [1, 4, 42–44]. For example, Deepvessel [44] first applied a multiscale CNN to learn rich hierarchical features, and then combined conditional random fields to improve the performance of retinal vessel segmentation. SCS-Net [22] proposed three new modules to capture multiscale contextual information and promote the fusion of the features at different levels. DNL-Net [45] optimized the structure of the non-local block (NL) and presented a deformed non-local neural network (DNL-Net) for retinal vessel segmentation. ResDO-UNet [46] proposed an automatic and end-to-end detection scheme from fundus images to enhance feature extraction capabilities. Kim et al. [47] adopted the concept of iterative learning in a U-Net-like model for medical image segmentation. Dasgupta et al. [14] used FCN to segment retinal vessels in color fundus photography images. R2U-Net [48] proposed a recurrent convolutional neural network (RCNN) for medical image segmentation. The Dense U-Net [49] transforms the convolutional modules in the common U-Net model into the dense block. SA-Net [50] proposes a scale of attention to enforce the scale-attention capability, which can learn the multiscale features and improve accurate segmentation.

Although the above-mentioned achieved notable performance, these algorithms still have some problems with feature information retention. Specifically, the existing methods suffer from the following limitations. First, these semantic segmentation models usually only use the output of the last layer of the final decoding stage as the predicted segmentation map. In principle, the output of each layer in the decoding stage contains abundant segmentation information. Second, the vanilla skip connections are used to connect high-level features and shadow features in the U-shaped based structures network. However, shallow features that have undergone subsampling and convolution operations can suffer from information loss. Therefore, to address the shortcomings mentioned above, we redesigned the encoder-decoder structure. Compared with the traditional encoder-decoder structure, SFA-Net can better reduce the information loss in the coding and decoding stages and improve the performance of retinal vessel segmentation.

## 3. Method

**Figure 2:**
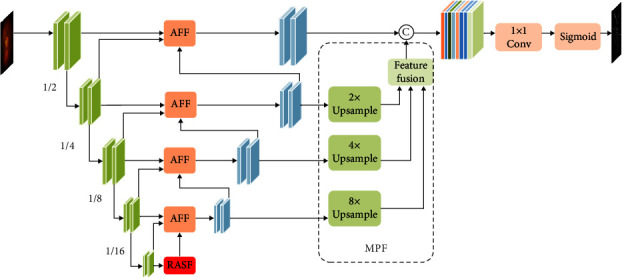
The SFA-Net architecture with convolutional encoding and decoding units consists of three main blocks: RASF, AFF, and MPF. © denote the feature connection.

The proposed SFA-Net, which is an encoder-decoder structure, is shown in Figure 2. We optimize both the coding and decoding layers of the U-Net for retinal vessel segmentation. In the coding layer, we use a new structure for feature extraction, and in the decoding layer, three different modules (RASF, AFF, and MPF) are proposed for the extraction of multiscale features and increasing the network perceptual field. The details are explained.

In the coding phase, we used the “Conv + BN” module, which is a simple convolution and BN module:

(1) $$\begin{array}{c} F\left(x\right)=Relu\left(BN\left(conv\left(x\right)\right)\right)\text{,} \end{array}$$

where BN represents a batch normalization and ReLu represents a rectified linear unit (ReLu).

In the decoding phase, the RASF block consists of residual atrous networks, where the dilated convolution rates are set to 1, 3 and 5. The AFF module is designed to combine high-level features information with shallow features information. The MPF module is used for learning global semantic feature information from each layer of the decoder phase.

### 3.1. Residual Atrous Spatial Feature Aggregate (RASF)

**Figure 3:**
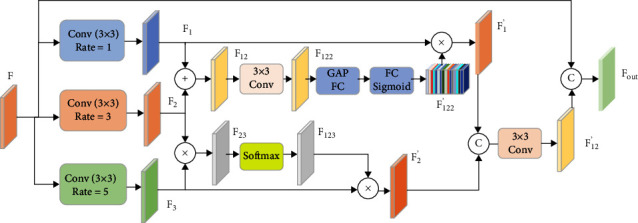
The diagram of the RASF block. ⊕ and ⊗ denote the tensor summation and multiplication. © denote the feature connection. Note that GAP means global average pooling and FC means fully connected.

The receptive field is important in semantic segmentation. A number of scholars have proposed methods to increase network segmentation accuracy by increasing the receptive field, such as Fu et al. [51], Shi et al. [42], Tao [52], and Larsson et al. [53]. Although these methods differ in structure and results, they all increase the network receptive field by using multiscale information. To follow this idea, we propose a residual atrous spatial feature aggregate (RASF) module as shown in Figure 3.

In the RASF module, the input feature map *F* ∈ *ℝ*^*C*_*in*_×*H*×*W*^, which is extracted from the encoder stage. First, the feature *F* be feed into three parallel atrous convolution layers with three dilate rates are 1, 3, and 5. After that, we obtained three new feature maps (*F*_1_, *F*_2_, and *F*_3_). Then, to preserve multiscale feature information, the feature maps *F*_1_, *F*_2_, and *F*_3_ were concatenated:

(2) $$\begin{array}{c} F_{12}=F_{1}⊕F_{2}\text{,}\\ F_{23}=F_{2}⊗F_{3}\text{,} \end{array}$$

where “⊕” denotes the tensor summation operation, ⊗ denotes the element-wise multiplication operation.

Considering the problem of feature redundancy and suppressing the irrelevant background noise, we introduce an attention mechanism to automatically select the importance of the feature map. We fused the *F*_12_ by employing a 3 × 3 convolution, and then obtained the feature maps *F*_122_: *F*_122_=*δ*(*f*(*F*_12_)). Where *f*(·) function is the convolution with a kernel size of 3 × 3; *δ*(·) represent activation function (ReLU). After the convolution, the global average pooling (GAP) is used to extract the global feature information. Then, the feature map *F*_122_′ is obtained after two full connected operations and Sigmoid activation operations. Then, we will perform a matrix multiplication operation on the feature map *F*_122_′ and the feature map *F*_1_ to obtain the feature map *F*_1_′. In addition, we send the feature map *F*_23_ to the Softmax operation. After the Softmax operation, the feature map *F*_123_ and the feature map *F*_3_ are subjected to a matrix multiplication operation to obtain the feature map *F*_2_′. Then, we combine the feature map *F*_2_′ and the feature map *F*_1_′, and then use the 3 × 3 convolution to perform the dimensionality reduction operation. Finally, the feature map *F*_12_′ and the feature map *F* are aggregated via a residual connection.

### 3.2. Attention Feature Fusion (AFF)

**Figure 4:**
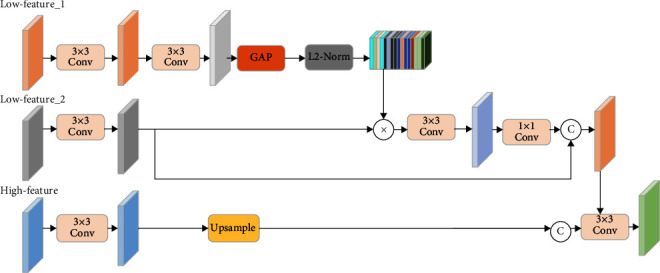
The flowchart of the AFF block.

To make better use of the feature maps in the coding and decoding stages, U-Net immediately concatenates the high-level and shadow features by employing vanilla skip connections. However, this connection ignores shadow features, and high-level features contain different feature information at different stages. High-level features contain more semantic information than shadow features but often lack spatial information. Low-level features have much more spatial information, which helps reconstruct intricate details. In addition, multiple convolution and subsampling operations in the encoding stage may lead to the loss of feature information. Hereby, we fuse low-level features before and after subsampling in the attention feature fusion (AFF) module to prevent the loss of information, as illustrated in Figure 4.

We first perform a global average pooling (GAP) operation on the low-level features_1 and perform a matrix multiplication operation with the low-level features_2. This operation can effectively suppress the irrelevant background noise in the shadow features. Then, we perform a transpose convolution operation on the high-level features to recover the resolution of the high-level features. Finally, fusing the shallow features and the high-level produces the final output features as follows:

(3) $$\begin{array}{c} F_{out}=F_{re}©f\;\left(F_{hig}\right)\text{,}\\ F_{re}=F_{low2}©F_{newlow}\text{,}\\ F_{newlow}=\delta \left(GAP\left(f^{\prime }\left(F_{low1}\right)\right)\right)\times F_{low2,} \end{array}$$

where *f* and *f*′ denotes the transpose convolution layer and 3 × 3 convolution;  ©  and *δ* means the concatenate operation and *L*2-Norm; GAP is global average pooling operation.

### 3.3. Multi-Path Feature Fusion (MPF)

**Figure 5:**
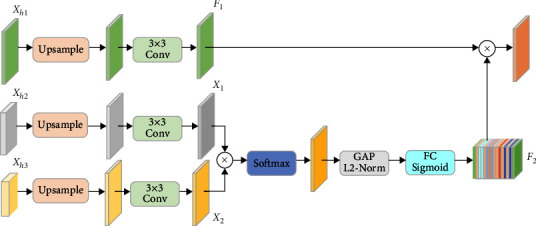
Schematic diagram of the MPF block.

In order to obtain more semantic information and improve network accuracy, we introduce the MPF module to improve the efficiency of the network as shown in Figure 5. Specifically, we used an attention mechanism to fuse the high-level features of the different stages. In the classical code-and-decode structure, the final output of the high-level features is used as the segmentation prediction result. However, the multiple upsampling and convolution operations usually result in the loss of high-level information. Therefore, we used the high-level features from different stages of the decoding phase for the final segmentation prediction.

We first perform upsampling operations on the high-level features at different stages to recover the resolution of the features. Then, we perform a convolution operation on the high-level features to optimize the high-level features. Also, we use a self-attention-like operation to perform feature filtering and reduce redundant features. Finally, a matrix multiplication operation is performed to fuse the high-level features map:

(4) $$\begin{array}{c} F_{out}=F_{1}⊗F_{2}\text{,}\\ F_{1}=Conv_{3\times 3}\left(Upsample\left(X_{h1}\right)\right)\text{,}\\ F_{2}=\delta \left(GAP\left(sofmax\left(X_{1}⊗X_{2}\right)\right)\right)\text{,}\\ X_{1}=Conv_{3\times 3}\left(Upsample\left(X_{h2}\right)\right)\text{,}\\ X_{2}=Conv_{3\times 3}\left(Upsample\left(X_{h3}\right)\right)\text{.} \end{array}$$

### 3.4. Loss Function

In this paper, considering the distributions of foreground and background in the retinal vessel map, we employ the binary cross entropy for training SFA-Net, defined as follows:

(5) $$\begin{array}{c} L=-y_{i}\log\left(p_{i}\right)-\left(1-y_{i}\right)\log\;\left(1-p_{i}\right)\text{,} \end{array}$$

where *y*_*i*_ and *p*_*i*_ represent ground truth and predicted probability, respectively.

(6) $$\begin{array}{c} p_{i}\in \left[0,1\right]\text{,}\\ y_{i}\in 0,1\text{.} \end{array}$$

## 4. Results and Discussion

### 4.1. Datasets Summary and Experimental Details

**Figure 6:**
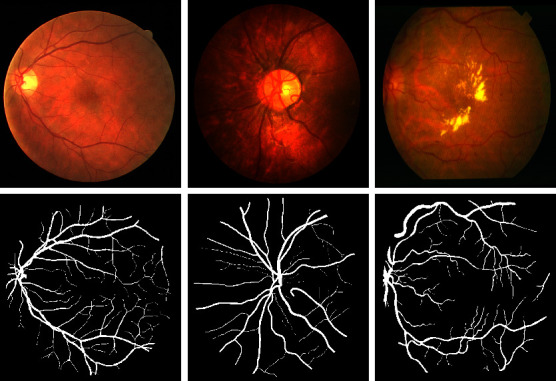
Example images from the training dataset, from left to right are DRIVE dataset, CHASEDB1 dataset, and STARE dataset. The first row shows the original images, the second row shows the label.

Three public datasets (DRIVE, CHASEDB1, and STARE) were used to test our model as shown in Table 1. All the images are RGB color in various formats and sizes, as illustrated in Figure 6.

The DRIVE (Digital Retinal Images for Vessel Extraction) dataset [54] consists of 40 color retinal images with a resolution of 565×584. The dataset is already divided into test and training, with 20 samples utilized for training and the remaining 20 samples for testing.

The CHASE_DB1 (Child Heart and Health Study in England) dataset [55] contains 28 retinal images, and the size of each image is 999 × 960 pixels. The dataset is divided into two sets, in which a 20-sample set is used for training and the remaining 8 samples are used for testing.

The STARE (Structured Analysis of the Retina) dataset [56] contains 20 colorful retinal fundus images, and each image has a size of 700 × 605 pixels. We employed the leave-one-out strategy on the STARE dataset due to the smaller number of samples, which means that one image is used for testing and the remaining 19 samples are used for training.

We used 190,000 randomly chosen patches from the retinal blood vessel images in all three datasets. In this implementation, 152,000 patches were used for training, and another 38,000 patches were used for validation. For all three datasets, the patch size is 96 × 96. The Keras platform and an NVIDIA TITAN XP graphics card with 12 GB of memory are used to implement our proposed SFA-Net. After repeating the upsampling operation and down-sampling operation four times, the number of train parameters is 9.3 M. In addition, we employ the Adam as an optimization approach during training, with an initialization learning rate of 1*e* − 4 and a weight decay of 0.0001. The batch size in our experiments is set to 16.

Several significant metrics are used to quantitatively evaluate the experimental data, including sensitivity (SE), specificity (SP), and accuracy (ACC), which are calculated using the following:

(7) $$\begin{array}{c} SE=\frac{\left|TP\right|}{\left|TP+FN\right|}\text{,}\\ SP=\frac{\left|TN\right|}{\left|FP+TN\right|}\text{,}\\ ACC=\frac{\left|TP+TN\right|}{\left|TP+TN+FN+FP\right|}\text{,} \end{array}$$

where TP and FP are the true positive and false positive, respectively. Correspondingly, TN is the true negative, while FN is the false negative. The receiver operating characteristic curve (ROC) area under the curve (AUC) is also used to evaluate segmentation performance, which is dependent on recall and precision.

### 4.2. Results

**Figure 7:**
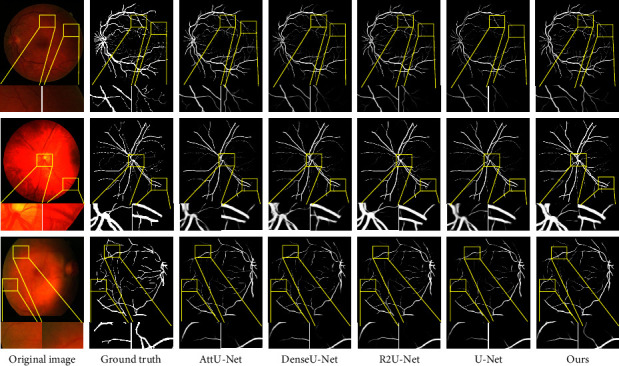
Example segmentation results on three databases: DRIVE (top), CHASEDB1 (middle), STARE (bottom).

To demonstrate the outstanding performance of our proposed method, we compare it to other state-of-the-art deep learning methods, such as U-Net [16], R2U-Net [48], AttU-Net [23], and DenseU-Net [49]. We implemented all algorithms on three datasets (DRIVE, CHASEDB1, and STARE). The experimental result indicators for these methods are shown in Table 2 and Figure 7.

We used four significant statistical metrics (SE, SP, ACC, and AUC) to analyse the SFA-Net on three public datasets. As shown at the top of Table 2, compared with the other four algorithms, SFA-Net shows better performance than the state-of-the-art methods in most metrics on the DRIVE dataset. In the DRIVE dataset, it has an AUC of 98.44%. It also has the highest SE of 83.53%, the highest ACC of 96.31%, and a SP of 98.09%. This means that our proposed SFA-Net is beneficial to retinal vessel segmentation. Also, in the CHASEDB1 dataset, with comparative results of other methods presented in the middle of Table 2. SFA-Net outperforms other methods in SE and ACC by 84.26% and 97.48%, respectively. When compared with AttU-Net, the SE increased from 77.87% to 84.26%. The ACC and AUC increased from 96.36%/98.29% to 97.48%/98.93%, respectively. The STARE dataset performance comparisons are shown at the bottom of Table 2. In terms of AUC evaluation metrics, SFA-Net outperforms other algorithms, and it also outperforms other algorithms in terms of SE and ACC.

**Figure 8:**
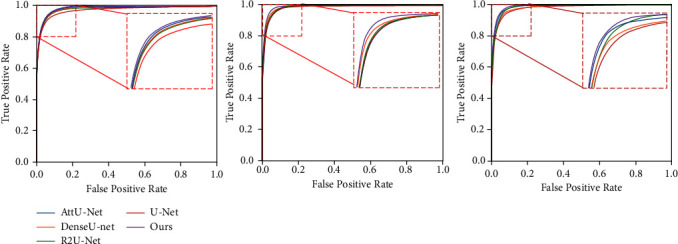
For retinal vessel segmentation, the ROC curves of various models are shown. From left to right, DRIVE, CHASEDB1, and STARE.

In addition, we utilize ROC curves and AUC metrics to evaluate the performance of the SFA-Net, as shown in Figure 8. We can see that the SFA-Net outperforms state-of-the-art methods by obtaining the highest ROC and AUC values.

### 4.3. Ablation Studies

**Figure 9:**
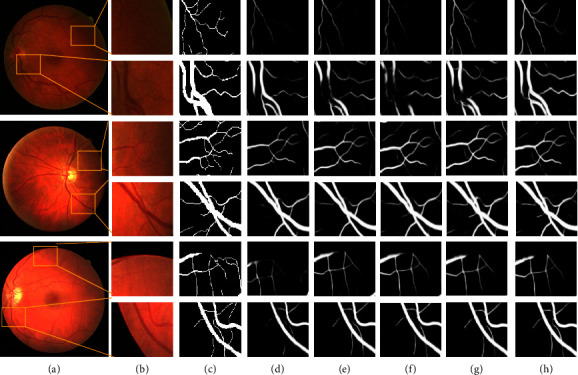
Example segmentation results of different blocks in ablation research on the DRIVE dataset. (a) Original image, (b) detailed view, (c) ground truths, (d) Baseline, (e) Baseline + RASF, (f) Baseline + AFF, (g) Baseline + RASF + AFF, and (h) Baseline + RASF + AFF + MPF (Ours).

The SFA-Net contains three main components in the proposed approach in order to illustrate that each block of the SFA-Net can improve the performance of retinal vessel segmentation. We implement ablation research to prove the performance of each block on the DRIVE database.  Figure 9 and Table 3 show the visual results components and statistical comparisons, respectively. A U-shaped network (U-Net) is referred to as “Baseline” in ablation study.

#### 4.3.1. Effectiveness of the RASF Module

The Baseline with the RASF module is referred to as “Baseline + RASF.” Compared with “Baseline,” “Baseline + RASF' increases from 73.24% to 79.84% in terms of SE. The ACC and AUC increased from 95.63% to 97.06% to 96.20% and 98.28%, respectively. In addition, we add the RASF block into the “Baseline + AFF,” which is referred to as “Baseline + RASF + AFF.” Compared with “Baseline + AFF,” the performance of “Baseline + RASF + AFF” increases by 0.3% in terms of SE. This means that our proposed RASF module is beneficial for effectively obtaining multiscale information and improving network accuracy.

#### 4.3.2. Effectiveness of the AFF Module

The Baseline with AFF module is referred to as “Baseline + AFF.” Compared with the “Baseline,” the “Baseline + AFF” increases in terms of SE by 12.5% (from 73.24% to 82.42%). Then, we further embed AFF into “Baseline + RASF” (referred to as ‘Baseline + RASF + AFF') to further enhance the validity of the AFF module. As shown in Table 3, the AUC and SE of “Baseline + RASF + AFF” are 0.12% and 0.08% better than the “Baseline + RASF network.”

#### 4.3.3. Effectiveness of the MPF Module

In order to learn multilevel features, MPF is inserted into different stages of the decoding phase. To verify its validity, we add the MPF block into the ‘Baseline + RASF + AFF(referred to as “Baseline + RASF + AFF + MPF (ours)”). Compared with the ‘Baseline + RASF + AFF', the proposed SAT-Net has an increase in AUC of 0.65%, as shown in Table 3. This means that the proposed MPF module is effective at the decoder stage and improves network accuracy.

### 4.4. Limitations

Currently, the main retinal vessel segmentation algorithms are based on convolutional neural networks, which often require a large amount of data during training. Since the images of fundus vascular datasets are usually small, resulting in the robust performance of the trained models being poor. However, traditional machine learning methods are usually better at dealing with small data sets. Therefore, we should pay more attention to the combination of traditional machine learning methods with convolutional neural networks to improve the accuracy of retinal vessel segmentation.

In our experiments, we find that when the contrast between the blood vessels and the background noise is low in the retinal vessel pictures, there are still some failure cases as shown in Figure 7. The reason for this situation is that we believe that the fundus vascular dataset is small and it is difficult for the model to learn rich features. However, even under such conditions, our method outperforms other state-of-the-art methods, and our model's segmentation results are more accurate.

## 5. Conclusion and Future Works

This paper proposed a retinal vessel segmentation network (SFA-Net) with three module components, i.e., RASF, AFF, and MPF. The proposed method can effectively capture multiscale contextual information and fuse high-level and shadow features to improve network segmentation accuracy. The RASF module is designed to feature aggregate, which uses dilated convolution to dynamically change the receptive field and learn more multiscale contextual information. Besides, the AFF modules fuse shadow and high-level features in each stage of the encoding layer. In addition, the MPF involves all shallow features at different scales in the coding stage in the final feature segmentation to improve the network segmentation accuracy. The experimental results demonstrate the outstanding performance of the SAF-Net in the segmentation of retinal vessels.

In the future, we will introduce traditional machine learning methods into deep learning to improve semantic segmentation accuracy. Although various deep learning-based semantic segmentation algorithms have achieved excellent results, they do not perform well when dealing with datasets with a small number of images. We will introduce traditional machine learning methods to improve the ability of deep learning to deal with datasets with a small number of images.

## Figures and Tables

**Table 1:** An overview of the three publicly available retinal vessel databases.

Dataset	Quantity	Train-test split	Resolution	Format
DRIVE	40	20–20	565 × 584	.Tiff
CHASEDB1	28	20–8	999 × 960	.Jpg
STARE	20	18–2	400 × 605	.Ppm

**Table 2:** Experimental results of SFA-Net for segmentation and comparison with other approaches on three retina vessel databases: DRIVE, CHASEDB1, and STARE.

Method	SE (%)	SP (%)	ACC (%)	AUC (%)
DRIVE dataset				
Dense U-Net	80.40	98.23	96.04	97.97
AttU-Net	83.19	97.87	96.07	98.11
R2U-Net	83.18	97.71	95.93	98.01
U-Net	73.24	98.76	95.63	97.06
SFA-Net (ours)	83.53	98.09	96.31	98.44

CHASEDB1 dataset				
Dense U-Net	80.63	98.51	97.38	98.66
AttU-Net	77.87	98.23	96.36	98.29
R2U-Net	82.23	97.85	96.42	98.38
U-Net	79.97	98.05	96.39	98.33
SFA-Net (ours)	84.26	98.36	97.48	98.93

STARE dataset				
Dense U-Net	68.34	99.06	97.00	98.02
AttU-Net	71.09	99.00	97.13	98.51
R2U-Net	73.06	98.60	96.29	98.51
U-Net	67.63	98.77	95.96	97.59
SFA-Net (ours)	76.47	98.82	97.32	98.84

**Table 3:** Ablation studies on the DRIVE dataset for each block.

Method	SE (%)	SP (%)	ACC (%)	AUC (%)
Baseline	73.24	98.76	95.63	97.06
Baseline + RASF	79.84	98.08	96.20	98.28
Baseline + AFF	82.42	98.08	96.16	98.22
Baseline + RASF + AFF	85.13	97.71	96.18	98.34
Baseline + RASF + AFF + MPF (ours)	83.53	98.09	96.31	98.44

## Data Availability

The data used to support the findings of this study have been deposited in the DRIVE, CHASE_DB1, and STARE repository, the dataset is freely available and can be downloaded from DRIVE (https://drive.grand-challenge.org/), CHASE_DB1 (https://blogs.kingston.ac.uk/retinal/chasedb1/), and STARE (http://cecas.clemson.edu/∼ahoover/stare/).
